# Different Models of Everyday Physical Activity and Occurrence of Falls Among Community-Dwelling Older Adults and Middle-Aged Citizens

**DOI:** 10.3390/jcm15145471

**Published:** 2026-07-13

**Authors:** Aleksandra Stronk, Anna Lipert

**Affiliations:** Cardiovascular and Metabolic Diseases Prevention Laboratory, Department of Preventive Medicine, Medical University of Lodz, 92-213 Lodz, Poland; aleksandra.stronk@stud.umed.lodz.pl

**Keywords:** WHO recommendation, falls, training, physical activity

## Abstract

**Background/Objectives**: The aim of this study was to assess the association between physical activity (PA), in accordance with the WHO recommendations for weekly activities, and the occurrence of falls over a one-year period. The study was conducted among 150 individuals aged 48 to 88 years living in the Polish city of Łódź during the Community Festive event. **Methods**: Data from 150 participants (131 women and 19 men) were assessed using the Tinetti scale, Time Up & Go (TUG), Hand Grip Strength (HGS), and a structured face-to-face interview regarding retrospective fall incidents over the past 12 months and the weekly volume of PA. **Results**: A total of 29 participants experienced a fall (all of whom were women) while 121 individuals reported no fall. In the non-fall group, higher percentage of individuals met the WHO physical activity guidelines compared to the fall group (74% vs. 61.1% to 48.3%). Furthermore, individuals without the history of falls achieved better results in the RHGS (*p* = 0.02) and Tinetti tests (*p* = 0.04). Notably, individuals in the fall group reported engaging in more aerobic activity on average than those in the non-fall group. **Conclusions**: The study demonstrated that older adults and middle-aged individuals without a history of falls reported a higher compliance with weekly resistance training. While multivariable models show that raw physical activity volume is not an independent predictor of fall risk, training compliance aligns with superior objective neuromuscular performance, showing positive associations with RHGS and Tinetti scores.

## 1. Introduction

The general availability of doctors, as well as improved living conditions, contributes to increased life expectancy aznd, above all, improved quality of life. The mere awareness of healthy lifestyles and preventive care allows people to live longer and healthier lives, which, in turn, increases the total number of people over the age of 60. According to the Central Statistical Office (GUS), at the end of 2022, the number of people aged 60+ exceeded 9.8 million. Compared to the previous year, this number was 0.7% lower. In Lodz, at the end of 2023, there were 240,700 people over 55 years of age and 166,100 over 65 years old. Currently, seniors constitute 25.9% of the population, and according to forecasts, this number is expected to increase by 30% by 2060. In Europe, however, the number of elderly people increased to 21% between 2002 and 2022 [1].

The aging process in the body is natural, irreversible, and progresses at varying rates. For several years, the dominant trend has been toward faster aging due to poor eating habits, reduced physical activity, increased stress, and substance use [2]. According to the “Dunedin Study,” which examined longitudinal measurement of telomeres, discrepancies between metric and biological age were demonstrated, as well as early symptoms of aging as early as the late third decade of life [2,3]. Furthermore, people with a higher biological age are aging faster. As a result of aging, the body’s adaptive capabilities deteriorate [4], which may result in the development of psychological problems, including social withdrawal, but also physical problems, including differences in endurance or the ability to participate in both sports and everyday activities, can arise.

Balance is the ability to maintain the body’s center of gravity, located in the lower abdomen, within the support surface defined by the contours of the feet [5]. The ability to determine this point by the patient provides a spatial reference point from which all other movements can be performed, as well as the ability to assess one’s condition and adapt to new situations [6]. The process of maintaining balance involves not only the musculoskeletal system, but also the nervous system, vision, hearing, and superficial and deep sensations [7,8]. Any changes occurring in these systems, combined with a decline in physical fitness, significantly increase the risk of falls and, consequently, injuries in people over 60. A fall (according to the WHO) is an event in which a person unintentionally finds themselves on the floor or other low surface due to a loss of balance, e.g., while walking [9]. Falls are among the so-called “Great Geriatric Problems” and are one of the most serious causes of disability in older adults [10]. Falls can significantly impact the quality of life of geriatric individuals, both through the development of Post-Fall Syndrome and through health impairment related to the incident itself. According to statistics conducted by the National Institute of Public Health—National Institute of Hygiene in 2017, among people over 65 years of age, 50- 67% of nursing home residents, 33% of those living independently, and 20% of hospitalized patients experience one fall per year [10]. Many risk factors can be prevented, but with unmodifiable factors such as age, the only way to do this is to support the patient and guide them towards appropriate treatment and therapies [11].

It is widely considered that regular physical activity is one of the factors influencing mental and physical well-being. According to the WHO, people over 65 should engage in both spontaneous and regular exercise [12]. These activities are designed to strengthen muscles, build balance, and increase body performance. They help prevent falls, which have many consequences. The phenomenon of falls among older adults, their etiology, and the identification of support needs for those who experience falls too frequently is a very current issue. Recent studies also inform that PA can reduce the risk of certain major chronic diseases and promote the health status [13,14].

We did not find many publications regarding the typical fulfillment of the WHO recommendations for weekly physical activity in correlation with the occurrence of falls using a dynamometer test. Therefore, the aim of the study was to analyze various models of physical activity undertaken by community-dwelling older and middle-aged adults in relation to the occurrence of falls in them. The aim of this study was to assess the association between physical activity (PA), in accordance with the WHO recommendations for weekly activities, and the occurrence of falls over a one-year period among community-dwelling older and middle-aged citizens. We hypothesized that community-dwelling middle-aged and older adults who meet the WHO guidelines and report a higher volume of physical activity would demonstrate superior outcomes in functional fitness and neuromuscular strength tests, which could be exploratorily linked to differences in retrospective fall frequency.

## 2. Materials and Methods

The study was conducted among 150 individuals (131 women and 19 men) aged 48 to 88 years (70.15 ± 6.9) in the city of Lodz Poland. These were residents of Łódź participating in the Senioralia cultural event in the city center. The study was conducted in May 2024.

Inclusion criteria for the study included: (a) being an adult; (b) maintained verbal and logical communication; (c) the ability to walk. The exclusion criteria for the study were: (a) young age; (b) failure to provide written consent to the study; (c) persons using a wheelchair.

Although the study primarily targeted older adults aged 60 and above, a small subgroup of middle-aged individuals (N = 6, aged 48–59 years) was included in the final sample. These participants were active attendees of the local community health events where the recruitment took place, and their exclusion would overlook valuable community engagement data. To ensure that their inclusion did not introduce chronological age bias or distort the generalizability of the findings to the senior population, a formal sensitivity analysis was performed by temporarily excluding these six participants. The analysis demonstrated absolutely no impact on the statistical significance levels, odd ratios, or the final conclusions of both univariate and multivariable models. Therefore, they were retained in the final cohort (N = 150) to preserve sample integrity.

The group was recruited from a public cultural event, a convenience sample of relatively active and socially engaged individuals. Written consent was obtained from every participant who wanted to participate in the study.

Questions regarding falls and level of physical activity were assessed retrospectively based on a face-to-face structured interview. A fall was explicitly defined to participants according to the WHO criteria as an event which results in a person coming to rest inadvertently on the ground or floor or other lower level [12]. Participants were asked to recall the exact number of such incidents occurring within the preceding 12 months. After that, PA level was quantified. The structured interview was modeled after the operational domains of standard physical activity questionnaires, tracking four specific modalities: aerobic, resistance, stretching, and balance training. For each domain, participants reported the average frequency (days per week) and duration (minutes per session) over a typical week to calculate the total weekly volume. The aerobic training was categorized into MIPA (Moderate Intensity Physical Activity) which corresponds to WHO duration thresholds of 150–300 min of activity and VIPA (Vigorous Intensity Physical Activity) which corresponds to WHO duration thresholds of 75–150 min of higher intensity. Respondents were also asked about other additional forms of activity that WHO in its guidelines indicates as achieving additional health benefits.

Assessment of postural stability and gait accuracy was performed using physical fit-ness tests commonly used in geriatric wards: the TimedUp&Go test and the Tinetti scale. To assess muscle strength, (1) the JAMAR dynamometer (Sammons Preston, Warrenville, IL, USA) test was used, and to determine physical fitness, the following tests were performed: (2) the Romberg test, (3) the TUG test, and (4) the Tinetti scale Part I.

A dynamometer is a device for measuring strength. There are many variants on the market, but the most commonly used is the Jamar Hydraulic Hand Dynamometer. The one used to perform the measurements for this research was SH5001 Hydraulic Hand Dynamometer (Saehan Corporation, Masan, Republic of Korea). The Hand Grip Strength test protocol involved the participant sitting in a chair with their back resting on the chair backrest, their feet on the ground, their upper limb on a table, their shoulder and elbow flexed to 90 degrees, and their hand with the dynamometer extending off the table. Each participant completed three trials with a 60 s interval between measurements. The highest of these two measurements was selected and recorded. The test was performed on both the right and left hands. The EWGSOP2 cutoff was used, with men < 27 kg and women < 16 kg [15]. Regarding hand selection, the literature suggests performing the test on the dominant hand. According to Bohannon [16], right-handed individuals experience a greater difference between the right and left hands than left-handed individuals; in this case, the differences are insignificant. The Romberg test is one of the simplest methods for assessing static balance [17]. It is performed to identify a specific impairment in patients with specific proprioceptive difficulties in order to intervene and improve treatment outcomes.

The TUG (Timed Up & Go) test is a broad test used to assess the risk of falls in older adults [18]. It was developed to assess several components, including: the ability to perform quick and efficient maneuvers, such as turning around a chair; dexterity, such as quickly rising from a chair; and dynamic balance, such as regaining balance after standing and sitting down in a chair [17]. It is used not only for diagnostic differentiation but also to assess overall fitness and dynamic balance. According to the guidelines of the National Chamber of Physiotherapists in Poland, the normal range for healthy adults is less than 10 s, and for older adults, less than 14 s; a result above 30 s is a premise for the use of assistive devices [19].

The Tinetti scale (Performance Oriented Mobility Assessment—POMA) consists of two parts: balance and gait. The first section of the study, which focused on balance, was used in this study. It included nine tasks, each requiring a chair. It is based on the examination of balance while sitting and standing, both in natural conditions and in a provocative test involving nudging with eyes open and closed, a 360-degree rotation, and sitting back down in a chair [20].

According to WHO recommendations, total weekly activity time should include 150–300 min of Moderate Iintensity Physical Activity (MIPA) or 75–150 min of Vigorous Intensity Physical Activity (VIPA). Additionally, strength training should be performed twice a week, aimed at strengthening major muscle groups. The recommendation also includes additional light physical activity for approximately 300 min per week or Moderate Intensity Physical Activity for 150 min per week. For older adults, a balance training session is added three times per week [12].

## 3. Statistical Analysis

Statistical analyses were performed using Statistical version 13.1 software (StatSoft, Tulsa, OK, USA). The descriptive statistics were presented as percentages for dichotomous variables, and chi2 test was used for the comparison of percentages. Continuous variables are presented as median and confidence intervals. The data were not normally distributed (Shapiro–Wilk test for normal distribution analysis), so the Mann–Whine test was used to analyze the differences between the groups. Significant differences were accepted for all analyses at the level of *p* < 0.05. To assess the independent association of physical activity indicators with the risk of falling, multivariable logistic regression analyses (logit models) were computed, with fall status as the binary dependent variable and chronological age included as a continuous covariate. Separate, parsimonious models were constructed for total physical activity volume, aerobic training, resistance training, balance exercises, stretching and other activities to avoid overfitting given the number of events (n = 29). Sex was excluded from the multivariable models due to a quasi-complete separation constraint, as no fall events occurred within the male subgroup, making standard maximum likelihood estimation mathematically unstable for this variable.

## 4. Results

### 4.1. Group Characteristic

The characteristics of the group are presented in Table 1. A numerical imbalance between women (n = 131) and men (n = 19) was observed. Regarding endurance training, the median value was Mdn = 300 (95% CI: 286.9–353.3) for the group of women (n = 131) and Mdn = 250 (95% CI: 187.2–328.6) for the group of men (n = 19). While this difference suggests a higher average activity level among women, the fact that women constituted the majority of the study population precludes drawing a definitive conclusion. For resistance training, balance exercises, stretching, and additional physical activity, the median value in both groups was Me = 0. This indicates that more than 50% of the study participants do not engage in these types of physical activity.

### 4.2. WHO Recommendations on Physical Activity and Falls

The group was divided into those with a fall in the past year (n = 29), and those without falls (n = 121). In Table 2 rows, there are numbers that presents how many people met the WHO recommendation in individual group. It was shown that 12.8% more respondents in the n = 121 group met the WHO recommendations for aerobic activity. A significant difference was observed in resistance training. In the (n = 29) group, only three individuals met the requirements, while in the (n = 121) group, only one in five individuals met the requirements. It was shown that 16 individuals (13.2%) in the group without falls combined moderate intensity exercise (MIPA) with strength training. No significant difference was observed in the performance of equivalent training between the groups. A higher percentage of individuals in the non-falling group also met the combined aerobic and resistance training requirements, along with additional weekly Vigorous Intensity Physical Activity (VIPA) activity.

#### WHO Recommendations on Physical Activity and Falls-Expension

The group was divided into individuals who had fallen in the past year (n = 29) and those who had not fallen (n = 121). The rows in Table 3 present the numbers that show how many people met the WHO recommendations in each group. It was shown that in the n = 121 group, 12.8% more respondents met the WHO recommendations for aerobic activity. Statistical analysis showed that individuals meeting the WHO guidelines for weekly exercise minutes of MIPA or VIPA (OR = 0.59; 95% CI: 0.26–1.34), RT (OR = 0.4; 95% CI: 0.11–1.43), MIPA and RT (OR = 0.23; 95% CI: 0.03–1.84), VIPA and RT (OR = 0.50; 95% CI: 0.06–4.20), and Additional 150 min of VIPA (OR = 0.45; 95% CI: 0.09–2.08) had a lower odds of falling, but the results were not statistically significant (*p* > 0.05). Due to the fact that each confidence interval (95% CI) contained the value of 1, the direction of the effect of a specific type of physical activity on the occurrence of a fall cannot be clearly determined. The results are probably due to too small a group of respondents.

### 4.3. Types of PA

The types of training performed alongside with comparison between the group with a fall (n = 29) and without a fall (n = 121) are presented in Table 4. Statistical analysis showed a significantly higher weekly average physical activity in the group (n = 121) in resistance training like in additional weekly activity (*p* < 0.05). Despite the fact that weekly average in MIPA or VIPA were higher in the group with falls, it was statistically significant (*p* < 0.05). The only result not statistically significant can be found in the balance exercise.

### 4.4. Standardized Test Results

The results of the tests and measurements of hand grip strength are presented in Table 5. Higher mean values in the n = 121 group were shown in the Right Hand Grip Strength (RHGS) (*p* = 0.02) and in the Tinetti scale part one (*p* = 0.04), where the results in both groups are high due to the overall high efficiency of the group. In the TUG test, despite the statistically insignificant results, it was shown that both in the group with a fall and without a fall the results were below the norm (n = 29, Mdn = 8, 95% CI: 8.0 to 9.3) (n = 121, Mdn = 9, 95% CI: 7.8 to 8.8), which for seniors is 14 s.

### 4.5. Standardized Test Results

To examine the independent associations of physical activity parameters with fall occurrence, six separate parsimonious multivariable logistic regression models were computed (Table 6). To fully control for key confounding factors, each regression model adjusted simultaneously for age, BMI and the total number of reported comorbidities. Biological sex was excluded from the multivariable matrix due to a quasi-complete separation constraint, as zero fall events were recorded within the male subgroup. As presented in Table 6, none of the individual physical activity modalities or the cumulative volume emerged as statistically significant independent pre-injury markers or predictors of fall status. In Model 1, aerobic training exhibited a marginal, non-significant descriptive trend. Across all six models, the contextual health covariates, such as age, body mass composition and comorbidity burden, consistently demonstrated statistically non-significant relationships with the dependent variable. This statistically uniform behavior explicitly indicates that within this sample, retrospective self-reported training volume does not predict independent fall safety variations once concurrent baseline health and demographic variables are statistically stabilized.

## 5. Discussion

Our study is one of a few examining the incidence of falls in older adults and middle-aged individuals and compliance with WHO physical activity recommendations. The aim was to assess guidelines adherence, the incidence of falls and objective functional performance metrics. Partly aligning with our initial assumptions, individuals in the non-fall cohort reported a higher frequency of weekly resistance exercises. However, the hypothesis of physical activity volume acting as an independent protective predictor could not be fully confirmed. Although, the non-fall group presented a higher percentage of individuals meeting the WHO guidelines for aerobic activity compared to the fall group, these differences did not maintain statistical independence when controlled for chronological age, BMI, and clinical comorbidities.

There are numerous publications on the types of exercise that reduce the incidence of falls. A review study by Sherrington [21] confirms that an appropriate training program can reduce the risk of falling in older adults by 23% (RaR 0.77, 95% CI 0.71 to 0.03). A 24% (RaR 0.76, 95% CI 0.70 to 0.82) reduction in the number of falls was shown when combining balance and functional exercises, and a 28% (RaR 0.72, 95% CI 0.56 to 0.93) reduction was observed when resistance training was added to the exercises program. Another review by Sherrington, combining several types of exercise, such as balance and resistance exercises, gives better results in preventing falls [22]. Our results show that individuals in the non-falling group (n = 121) engaged in more resistance training and additional physical activity. Patti’s randomized controlled trial [23] reported that Pilates, a group exercise program that included stretching and resistance training based on isometric exercises, reduced falls and improved stability in older adults. Combining these two types of training allows for body strengthening without the use of weights, making it an excellent solution for older adults, building a strong muscular core, which indirectly influences better balance reactions. The OTAGO program, which also includes strengthening and balance exercises, has a positive impact on fall prevention. It was developed in New Zealand as a home-based training program aimed primarily at preventing falls among older adults. The program is based on balance and strength training (including 5 warm-up exercises) as well as resistance and locomotion exercises (exercises that strengthen the lower limbs) [24,25]. The OTAGO program, which also includes strengthening and balance exercises, has a positive impact on fall prevention. According to Yang’s research [26], 30–50 min of exercise three times a week reduces the number of fall incidents. The OTAGO program combines strength and balance exercises, which provide a foundation for developing balance. Our study revealed a small number of individuals regularly engaging in balance training: in the first group (n = 29), only two individuals, and in the second group (n = 121), five individuals, representing 4.6% of the entire study population. This accessible type of training, available in the form of videos, produces real improvements in fitness and should be recommended for older adults. Having that in mind, according to Rodrigues [27], falls can also occur during prescribed exercises. These errors stem from a failure to consider the patient’s physical and cognitive abilities. Therefore, exercises should be tailored to the specific individual, not necessarily rigidly following general recommendations. Our study provides a nuanced perspective on the relationship between weekly physical activity levels and the occurrence of falls among community-dwelling older and middle-aged individuals. In our univariate analyses, a clear descriptive trend emerged, showing that a higher percentage of individuals in the non-fall group adhered to the WHO physical activity recommendations, particularly for resistance training. However, when subjected to a more rigorous statistical approach using multivariable logistic regression adjusted for chronological age, none of the tested physical activity parameters emerged as a statistically significant independent linear predictor of fall status (*p* > 0.05). When subjected to multivariable controls that integrated chronological age, body mass index and clinical comorbidity counts, none of the assessed physical activity parameters maintained statistical significance. This lack of statistical independence strongly underscores that the raw weekly volume of physical activity cannot be confirmed as an independent protective factor or predictor of fall risk in this population. The prominent descriptive differences observed in our initial univariate profiles (Table 4) are highly likely to be co-dependent and confounded by age-associated physical shifts, variations in baseline metabolic fitness and unmeasured clinical covariates. Because our fully adjusted multivariable equations yielded non-significant parameters, any deterministic or causal assumptions regarding training duration must be firmly rejected. These results demonstrate that physical activity levels operate within a web of broader physiological and health status determinants common among community-dwelling older adults. These results demonstrate that physical activity levels operate within a web of broader physiological and health status determinants common among community-dwelling older adults. This interpretation aligns with our objective clinical measurements; individuals in the non-fall group achieved significantly superior results in the Right Hand Grip Strength (*p* = 0.02) and the Tinetti balance tests (*p* = 0.04). Consequently, regular physical activity may be viewed as a vital upstream catalyst that mitigates fall risk indirectly by preserving neuromuscular strength and enhancing postural stability, rather than acting as a direct standalone linear preventer.

The study found that 29 of the 150 participants experienced a fall. All of them being woman. Due to the small sample size of men, it is difficult to determine whether falls occur more frequently in women rather than in men. According to Melzner’s study conducted in several countries, it was shown that women who fell were 61.0% and 39.0% for men [28]. However, there is a study examining the burden of falls solely in China over a 29-year period and their study found that there was no difference between gender and the occurrence of falls [29]. There is a need to conduct a study gathering equal numbers of women and men to check falls in the population and also programs for the prevention of falls.

Physical activity is great for your health but when you do it wrong it can impact you in negative way such as injuries or falls. As reported by our results in MIPA or VIPA, the average walking time per week in the group (n = 29) was greater than in the group (n = 121). This may reflect reverse causation, misclassification, recall bias, and rehabilitation after a fall, which increased awareness of the priority of movement to prevent falls. Increased activity may exacerbate falls due to inadequate infrastructure for sports or simply the greater risk of injury during movement than during passive leisure time.

Physical activity levels among older people are very dependable on where they live. A study conducted in the United States found that people living in rural areas had a lower percentage of adherence to guidelines for aerobic exercise, strengthening exercises, and a combination of both [30]. Older and middle-aged individuals living in cities have greater access to gyms and community centers offering group exercise classes. The study case that took place in Poland [31] confirms that people living in urban environment have lower volume of aerobic activity such a cycling, intense physical activity in garden areas and less time spent outdoor for walks. In opposition to previous statement, a study conducted in China [32] suggests that city dwellers are more willing to engage in physical activity than residents of smaller towns, while residents of both groups showed that lack of time due to working conditions and appropriate conditions for practicing sports limits their opportunities to practice sports. Overall, it all depends not only on the willingness and involvement of residents, but also on architectural barriers and work systems that do not make it easier for residents to live actively. In both groups, balance training was not popular. In the group (n = 29), only two people, and in the group (n = 121), five people, met the WHO recommendations for balance training. Despite strong evidence of its protective effect on falls [30,31], group classes for older adults in Poland are based on group Nordic walking, general aerobic training, or weight training. Pilates and Tai Chi are slowly becoming more popular in Poland. With this in mind, research is needed to determine whether and over what timeframe these trainings provide benefits to Polish residents.

The strength of the study was the use of standardized balance assessment tests, i.e., the Tinetti test, and the use of a dynamometer providing objective results of measuring muscle strength in seniors. These tests are of great clinical importance, enabling accurate description and comparison of results, ensuring ease and reliability in conducting future tests of a similar social group. A group of seniors in real-life environment assessed their activity levels, whether they adhered to the recommendations, and whether they had experienced falls. The adopted strategy allowed for comparison outside the hospital setting, providing a group. This provides an overview of the overall average physical activity in society. The study itself allowed us to further educate participants about physical activity, how much they should engage in, and to suggest sample exercises to do at home. Such recommendations should be provided and made known to older people by healthcare professionals or in events like this.

A limitation of the study is the convenience sampling from a senior event, where the group is diverse but limited to individuals active in social life. The significant gender imbalance and the inclusion of participants other than those typically defined as seniors prevent us from drawing clear conclusions and only provide guidance. Another major limitation is the lack of adjustment for critical clinical confounders, such as visual acuity impairments, neurological conditions or fall-risk-inducing medications which were not quantified in this study. Furthermore, while the total cohort was relatively stable, the small absolute number of fall events constrained our statistical power, preventing the inclusion of multiple concurrent covariates in a single regression model without a severe risk of overfitting. Consequently, our multivariable findings must be strictly interpreted as exploratory trends rather than definitive confirmatory parameters. Regarding the survey, self-assessment of activity, history of falls, and more detailed information about these events limit a broader interpretation of the results. The lack of generalizability of the results and the potential ceiling effect only allow interpretations for a narrow group of respondents.

## 6. Conclusions

The presented study allows us to conclude that community-dwelling older adults and middle-aged individuals without a retrospective history of falls were more likely to report participation engagement in weekly resistance training. However, because physical activity volume did not emerge as an independent predictor of fall status, regular training should be conceptualized as an upstream catalyst that aligns with superior objective neuromuscular and functional metrics rather than a direct, standalone linear preventive factor. Physical activity should be further promoted among older and middle-aged people through a variety of local events and the implementation of activity programs in clinics, nursing homes and community centers. It is recommended to promote physical activity as a key to well-being and independence. Further research involving a larger and more diverse group of older and middle-aged people, including those in rural settings, is recommended.

## Figures and Tables

**Table 1:** Characteristics of the group by gender.

	Woman (n = 131)	Man (n = 19)	All (n = 150)
Age Median (95% Cl)	70 (69.2–71.6)	69 (67.5–73.5)	69.5 (69.4–71.6)
Height Median (95% Cl)	1.6 (1.6–1.6)	1.75 (1.7–1.76)	1.6 (1.6–1.62)
Weight Median (95% Cl)	68 (66.6–70.4)	82 (75.3–89.9)	70 (68.4–72.4)
BMI Median (95% Cl)	26.3 (26.1–27.3)	25.9 (25.5–29.5)	26.0 (26.2–27.4)
Number of comorbidities Median (95% Cl)	1 (1.3–1.7)	2 (1.1–1.9)	1 (1.3–1.7)
Endurance Training Median (95% Cl)	300 (286.9–353.3)	250 (187.2–328.6)	300 (282.5–343.1)
Resistance Training Median (95% Cl)	0 (14.6–31.6)	0 (5.3–49.5)	0 (15.7–31.5)
Balance Training Median (95% Cl)	0 (11.8–27.2)	0 (−3.4–9.8)	0 (10.7–24.3)
Stretching Training Median (95% Cl)	0 (19.6–39.1)	0 (2.3–34.5)	0 (20–36.8)
Additional PA Median (95% Cl)	0 (33.8–66.6)	0 (14.8–122)	0 (36.9–68.1)

BMI—Body Mass Index, PA—Physical Activity.

**Table 2:** Number of people meeting WHO recommendations, divided by seniors with falls.

Type of PA	Fall (n = 29)	Without Fall (n = 121)	All (n = 150)
MIPA or VIPA	14 (48.3%)	74 (61.1%)	88 (58.7%)
MIPA and VIPA	10 (34.5%)	31 (25.6%)	41 (27.3%)
Resistance Training	3 (10%)	27 (22.3%)	30 (20%)
MIPA and RT	1 (3.4%)	16 (13.2%)	17 (11.3%)
VIPA and RT	1 (3.4%)	8 (6.6%)	9 (6%)
Balance Training	2 (6.9%)	5 (4.1%)	7 (4.7%)
Additional 300 min of MIPA	0 (0%)	1 (0.82%)	1 (0.7%)
Additional 150 min of VIPA	2 (6.9%)	17 (14%)	19 (12.6%)

MIPA—Moderate Intensity Physical Activity; VIPA—Vigorous Intensity Physical Activity; RT—Resistance Training.

**Table 3:** Number of people meeting WHO recommendations.

Variable	Fall (n = 29)	Without Fall (n = 121)	OR	95% CI	*p*-Value
Type of PA					
MIPA or VIPA Not met	14	74	0.59	0.26–1.34	0.21
	15	47	1.00	Ref.	-
MIPA and VIPA Not met	10	31	1.53	0.64–3.64	0.338
	19	90	1.00	Ref.	-
Resistance Training Not met	3	27	0.40	0.11–1.43	0.1591
	26	94	1.00	Ref.	-
MIPA and RT Not met	1	16	0.23	0.03–1.84	0.1681
	28	105	1.00	Ref.	-
VIPA and RT Not met	1	8	0.50	0.06–4.20	0.5269
	28	113	1.00	Ref.	-
Balance Training Not met	2	5	1.71	0.32–9.34	0.5306
	27	116	1.00	Ref.	-
Additional 300 min of MIPA Not met	0	1	1.36	0.05–34.28	0.8512
	29	120	1.00	Ref.	-
Additional 150 min of VIPA Not met	2	17	0.45	0.09–2.08	0.3091
	27	104	1.00	Ref.	-

MIPA—Moderate Intensity Physical Activity; VIPA—Vigorous Intensity Physical Activity; RT—Resistance Training.

**Table 4:** Characteristics of physical activity undertaken in groups of people with and without falls.

	Fall (n = 29)	Without Fall (n = 121)	*p*-Value
Resistance Training Median (95% Cl)	0 (3.4–31.4)	0 (15.9–34.3)	0.005 *
Balance Training Median (95% Cl)	0 (3.9–32.7)	0 (9.5–25.1)	0.87
MIPA or VIPA Median (95% Cl)	400 (293.8–431.0)	300 (266.9–334.7)	0.005 *
Additional PA Median (95% Cl)	0 (18.1–72.9)	0 (36.3–73.1)	0.01 *

MIPA—Moderate Intensity Physical Activity; VIPA—Vigorous Intensity Physical Activity; PA—Physical Activity; * *p* < 0.05.

**Table 5:** Hand Grip Strength, TUG and Tinetti test results divided by fall and without fall groups.

	Fall (n = 29)	Without Fall (n = 121)	*p*-Value
RHGS Median (95% Cl)	21 (20.6–26.7)	28 (26.3–29.3)	0.02 *
LHGS Median (95% Cl)	22 (19.1–24.9)	23 (23.6–28.1)	0.07
TUG Median (95% Cl)	9 (8.0–9.3)	8 (7.8–8.8)	0.80
Tinetti Median (95% Cl)	15 (13.9–15.4)	16 (15.1–15.6)	0.04 *

RHGS—Right Hand Grip Strength; LHGS—Left Hand Grip Strength; TUG—Timed Up & Go; * *p* < 0.05.

**Table 6:** Multivariable logistic regression analysis of fall predictors (logit models).

Model Specification	Variable	OR	95% CI	*p*-Value
Model 1	Aerobic training	1.002	(1.000–1.004)	0.075
	Age	1.042	(0.976–1.112)	0.217
	BMI	1.063	(0.945–1.195)	0.306
	Number of comorbidities	1.094	(0.708–1.691)	0.686
Model 2	Resistance training	0.996	(0.987–1.006)	0.482
	Age	1.038	(0.973–1.106)	0.258
	BMI	1.037	(0.927–1.161)	0.525
	Number of comorbidities	1.089	(0.702–1.689)	0.705
Model 3	Balance training	1.000	(0.991–1.010)	0.927
	Age	1.038	(0.975–1.106)	0.244
	BMI	1.036	(0.925–1.159)	0.543
	Number of comorbidities	1.097	(0.709–1.698)	0.678
Model 4	Stretching training	1.001	(0.994–1.009)	0.774
	Age	1.038	(0.974–1.106)	0.251
	BMI	1.037	(0.926–1.161)	0.532
	Number of comorbidities	1.100	(0.709–1.706)	0.671
Model 5	Other types of physical activity	0.999	(0.994–1.004)	0.665
	Age	1.039	(0.975–1.107)	0.236
	BMI	1.035	(0.926–1.158)	0.544
	Number of comorbidities	1.090	(0.704–1.688)	0.699
Model 6	Total physical activity	1.001	(0.999–1.003)	0.268
	Age	1.039	(0.975–1.108)	0.241
	BMI	1.049	(0.934–1.178)	0.420
	Number of comorbidities	1.105	(0.714–1.712)	0.653

## Data Availability

The original contributions presented in this study are included in the article. Further inquiries can be directed to the corresponding author.

## Abbreviations

The following abbreviations are used in this manuscript:

MIPA	Moderate Intensity Physical Activity
VIPA	Vigorous Intensity Physical Activity
RHGS	Right Hand Grip Strength
LHGS	Left Hand Grip Strength
PA	Physical Activity
RT	Resistance Training
